# Non-surgical Management of Complex Refractory Pyoderma Gangrenosum With Negative Pressure Wound Therapy With Instillation

**DOI:** 10.7759/cureus.18951

**Published:** 2021-10-21

**Authors:** Frank G Lee, Ethan Song, Sean J Wallace, Thomas J Shaughnessy, Mamtha Raj, Robert Teixeira, Marshall G Miles, Randolph Wojcik Jr.

**Affiliations:** 1 Division of Plastic and Reconstructive Surgery, University of South Florida Morsani College of Medicine, Tampa, USA; 2 Division of Plastic and Reconstructive Surgery, Lehigh Valley Health Network, Allentown, USA; 3 Department of Physical Therapy and Wound Care, Lehigh Valley Health Network, Allentown, USA

**Keywords:** rheumatoid arthritis, mepitel, instillation, negative pressure wound therapy, pyoderma gangrenosum

## Abstract

Pyoderma gangrenosum (PG) is a rare skin disorder primarily treated with immunosuppression medication. We report a case of a large, chronic PG wound treated with adjunct negative pressure wound therapy with instillation and dwell time (NPWTi-d) using nonadherent dressing (Mepitel) and reticular open-cell foam with through holes (ROCF-CC) with positive outcomes. The patient was a 62-year-old female with rheumatoid arthritis, Hashimoto’s thyroiditis, lymphedema, and morbid obesity who presented with a 19.5 cm x 13.2 cm x 2.1 cm wound of three years duration on the right posterolateral lower extremity that successfully responded to a multimodality approach of immunosuppression and wound vac therapy. We conclude in our case that NPWTi-d with Mepitel and ROCF-CC enhanced the wound healing process, and we discuss NPWTi-d’s potential role and benefit as an adjunctive therapy option for chronic and poorly controlled PG on patients taking concurrent immunosuppression.

## Introduction

Pyoderma gangrenosum (PG) is a rare neutrophilic dermatosis that is associated with inflammatory bowel disease and rheumatoid arthritis (RA). PG presents initially as a tender nodule, plaque, or pustule that develops into a painful ulcer with undermined margins, violaceous borders, and surrounding erythema [1]. Accurate identification of PG is important, as debridement may worsen the lesion through a phenomenon called pathergy. Newer wound care techniques like negative pressure wound therapy with instillation and dwell time (NPWTi-d) using an irrigating solution have shown promising results in difficult-to-treat wounds [2-4]. Additionally, with advancements in dressing types including reticular open-cell foam with through holes (ROCF-CC, Veraflo cleanse choice, KCI) and nonadherent dressing (Mepitel), the choice of dressing should be considered. We report a positive effect of using NPWTi-d with ROCF-CC and Mepitel on a complex case of PG that was not healing on systemic immunosuppression only.

## Case presentation

**Figure 1:**
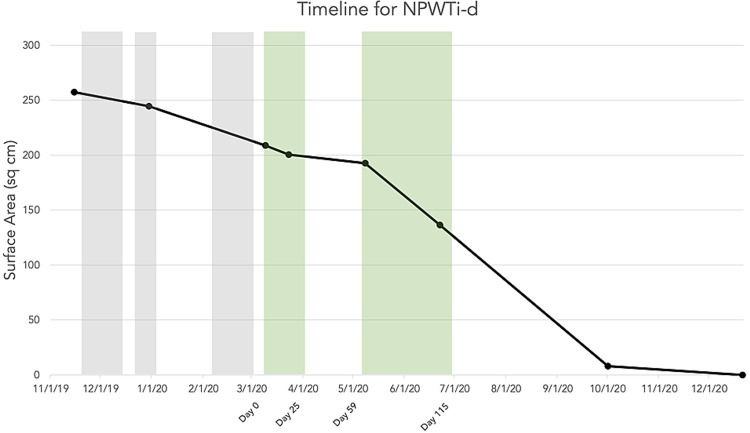
Progression of pyoderma gangrenosum wound surface area size vs time (gray denotes hospitalization periods without NPWTi-d, green denotes hospitalizations with NPWTi-d). NPWTi-d = negative pressure wound therapy with instillation and drainage

**Figure 2:**
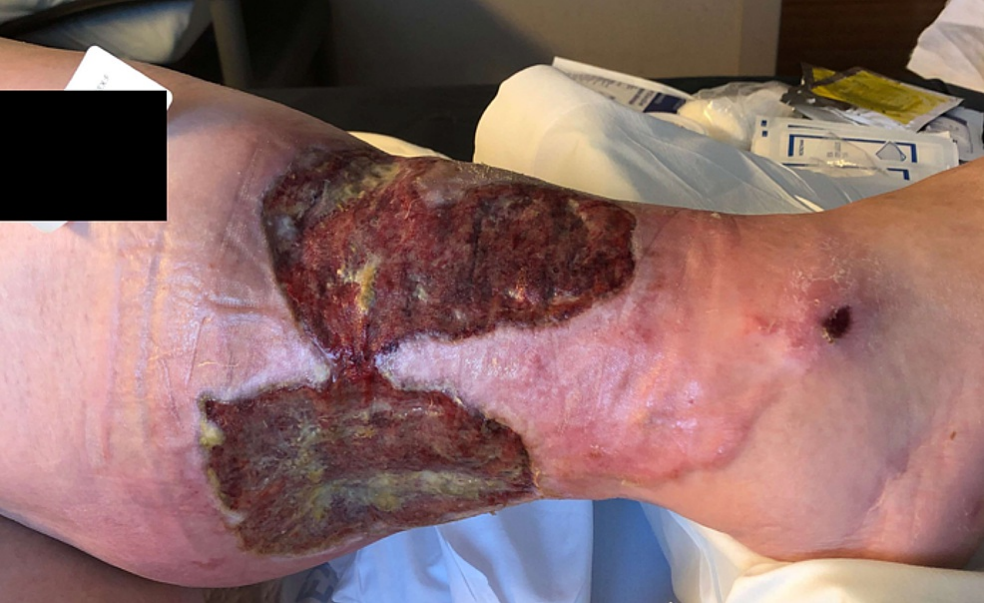
Chronic PG ulcer of three years on right posterolateral lower extremity prior to adjunct NPWTi-d therapy in addition to systemic immunosuppression therapy (day 0). Size is 19.7 x 10.6 cm. NPWTi-d = negative pressure wound therapy with instillation and drainage

**Figure 3:**
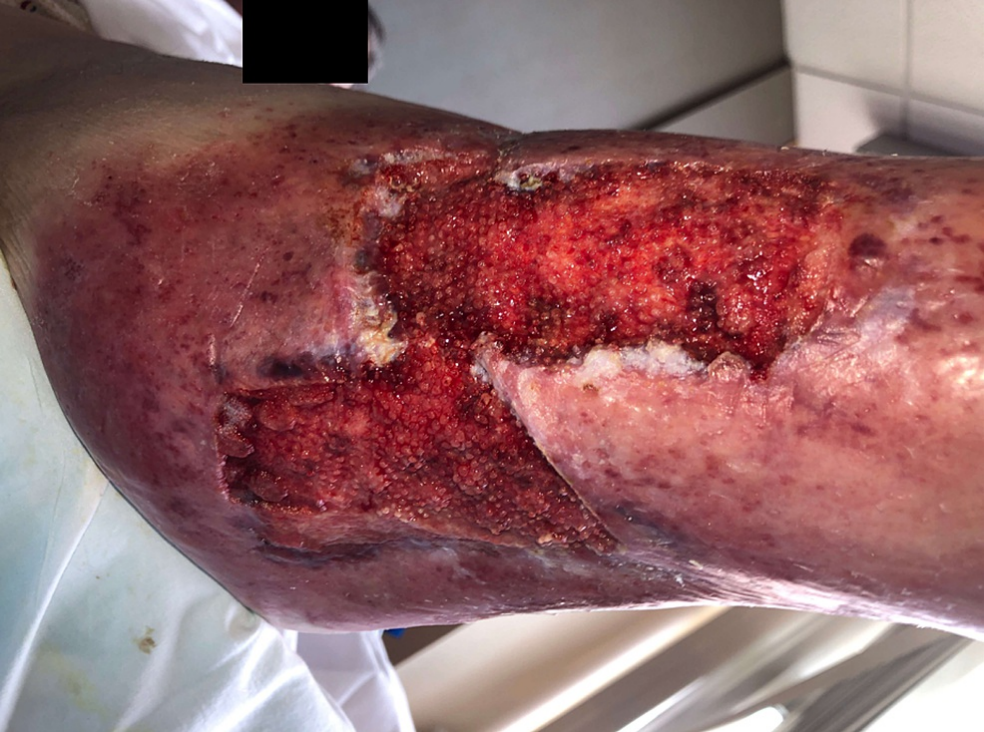
After 56 days of consecutive NPWTi-d therapy (115 days since the start of NPWTi-d). Size is 12.3 x 11.1 x 0.6 cm. NPWTi-d = negative pressure wound therapy with instillation and drainage

**Figure 4:**
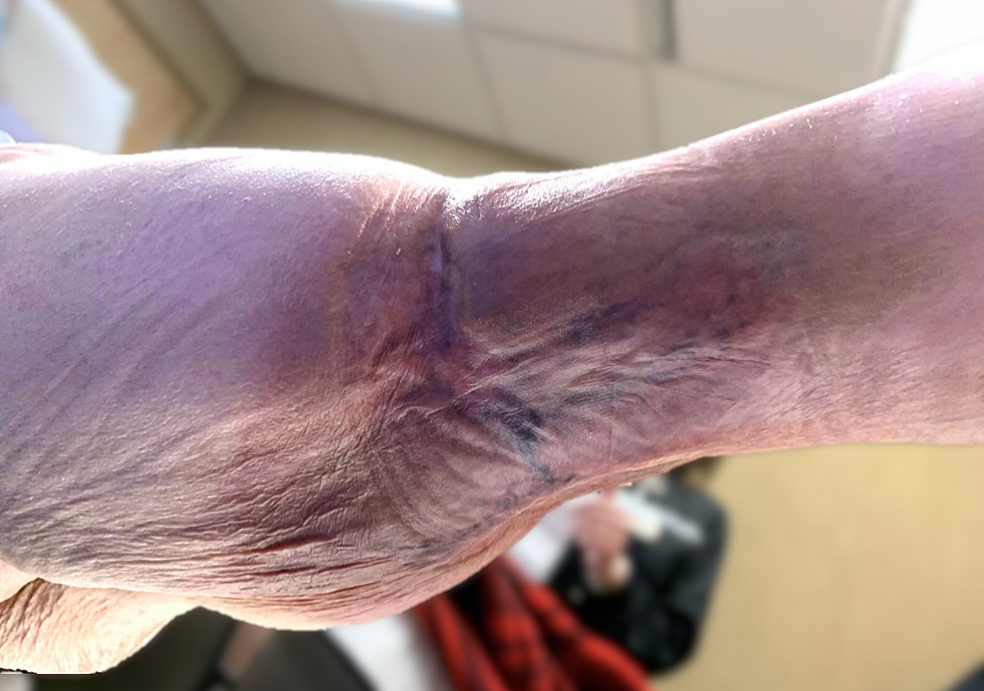
At the nine-month follow-up office visit, fully re-epithelialized and healed.

A 62-year-old female with RA, Hashimoto’s thyroiditis, lymphedema, fibromyalgia, and BMI 56 had a large chronic right lower extremity (RLE) wound for the past three years. She was diagnosed with pyoderma gangrenosum on biopsy which showed dense neutrophilic inflammation. Two years prior, she had stopped etanercept for her RA, and for the past year, she had not been to the lymphedema clinic (citing insurance difficulties for both). Skin grafts, including an Apligraft, were attempted three times at an outside facility and all of them failed. Over the next year, she was hospitalized multiple times for poor wound healing and superimposed infection, and treated with gentle debridement, standard vac therapy, and dressing changes with mupirocin and betamethasone. Rheumatology recommended prednisone during her hospitalizations of flare-ups, followed by maintenance methotrexate immunosuppression as an outpatient. Humira was attempted but discontinued due to diarrhea. After initial difficulty with adherence, a stable regimen of methotrexate 20 mg weekly with folic acid supplementation was established for the treatment of her RA. Due to the large size of the RLE wound and recurrent superimposed infections, her PG did not heal with methotrexate and wound care regimen. The PRS service decided to switch to NPWTi-d (Vac Veraflo) with 10 min dwell time, 3.5 hours between soaks, and negative pressure of 125 mmHg. Normal saline and dilute sodium hypochlorite (Dakin’s) solution were used for instillation. Wound care team noted significant improvement with Mepitel dressing as the contact layer on the wound bed, compared to Adaptic. Additionally, the patient preferred Mepitel reporting that it caused less pain during dressing changes. ROCF-CC was placed on top of the Mepitel. This set-up was used for the patient’s last two hospitalizations (Figure 1) from day 0 (Figure 2) to day 25, and readmission from day 59 to day 115 (Figure 3). On day 115, the patient was discharged with traditional NPWT. At her six-month outpatient follow-up visit, her wound was 4 cm x 2 cm. At nine months, the wound had completely healed (Figure 4).

 

 

 

.

## Discussion

In complex cases of PG, a multimodality approach of adjunctive wound vac therapy in combination with immunosuppression has yielded positive results for difficult-to-heal PG ulcers [2-7]. Due to concerns of pathergy, it is recommended that NPWT for PG be applied in the setting of adequate immunosuppression [8]. Traditional NPWT on PG has been well studied [5]; however, experience with the newer advanced technology of NPWTi-d on PG is limited. The newer technique of NPWTi-d which combines NPWT with instillation-and-dwelling of an irrigating solution over the wound environment has shown promising results for PG wounds complicated by infections [2]. Similarly, our case supports the use of NPWTi-d for complex refractory cases of PG with recurrent superimposed infections. We hypothesize that NPWTi-d’s mechanism of circulating solution is particularly effective in clearing the aberrant collection of neutrophils (characteristic of PG), which crowds out and blocks healing factors from reaching the wound site.

Without controlled trials on NPWTi-d on PG or other chronic and complicated wounds, limited high-quality evidence exists for the recommendation of NPWTi-d’s use in the management of difficult wounds. However, attempts at defining best practices for NPWTi-d have been made with the 2020 international expert consensus guidelines [9]. They recommend saline as the first choice for instillation solution followed by antiseptics, including sodium hypochlorite solution (dilute Dakin's solution 0.125% or quarter strength) as suitable alternatives, as used in our case. They also recommend ROCF sponge dressings with holes (ROCF-CC) to without holes (ROCF-V) for patients who are not candidates for sharp debridement, not indicated for PG. Additionally, the recommended parameters for NPWTi-d of 125 mmHg negative pressure, 10-minute dwell time, and two-to-three hours between treatment cycles align with that used for this patient.

While short-term use of NPWTi-d of one or two days has been proposed as a solution to reduce operating room time for washouts [10], we found that long-term use of NPWTi-d greater than 30 days was most effective in shrinking the size. Additionally, the preference for Mepitel over Adaptic was commented upon by both the wound care team and patient in providing greater comfort and appearance of the wound at dressing changes. A possible explanation is less biofilm adherence on the smoother Mepitel surface, which would reduce the risk of pathergy during dressing change. 

## Conclusions

Considering the unique challenges of this patient’s chronic presentation, complicated course, and large size of her PG wound, we report that adjunct use of NPWTi-d with Mepitel and ROCF-CC had a positive effect in resolving her PG wound. We purport the effectiveness of NPWTi-d may be due to its irrigative capability to clear the neutrophilic collection and allow passage of wound healing factors. Given the rare nature of PG, we hope that this case adds to the understanding of the non-operative approaches to PG and encourages further development of prospective studies to better elucidate outcomes of PG treated with NPWTi-d.

We report a complicated case of chronic PG on the RLE with positive outcomes using negative pressure wound therapy with instillation in adjunct with systemic immunosuppression. While the use of traditional NPWT is not new in PG, we present our experience with the newer NPWTi-d technology. We propose a multimodality approach should be considered in complex PG wound management.
